# Study on Rheological Behavior and Hot Processing Maps of a Novel Ultra-Low Carbon High-Strength Stainless Steel

**DOI:** 10.3390/ma19194088

**Published:** 2026-09-24

**Authors:** Dongchen Jiang, Renli Fu, Zhenbao Liu, Yuqiao Zhang, Wen Han, Chen Li, Xinquan Zhang, Jianxiong Liang, Zhiyong Yang, Wenyu Zhao, Peng Gao, Rui Wang, Jiarui Hu, Mutian Niu, Xiaohui Wang

**Affiliations:** 1 College of Materials Science and Technology, Nanjing University of Aeronautics and Astronautics, Nanjing 210016, China; woshijdc233@nuaa.edu.cn (D.J.);; 2 Division of Special Steels, Central Iron and Steel Research Institute, 76 Xue Yuan Nan Lu, Beijing 100081, China; liuzhenbao@nercast.com (Z.L.);; 3 Institute of Quantum and Sustainable Technology (IQST), School of Chemistry and Chemical Engineering, Jiangsu University, Zhenjiang 212013, China; yuqiaozhang@ujs.edu.cn; 4 Institute of Standard Process and Material Design, AVIC The First Aircraft Institute, Xi’an 710089, China

**Keywords:** high-strength stainless steel, hot processing map, hot deformation, dynamic recrystallization

## Abstract

This study investigated the hot deformation behavior of a novel ultra-low carbon high-strength stainless steel through single-pass isothermal hot compression tests using a Gleeble-3800 thermomechanical simulator. The hot deformation behavior were examined under deformation temperatures ranging from 900 to 1150 °C, true strains of 0.2 to 0.8, and strain rates of 0.01 to 10 s^−1^. The differences in recrystallization mechanisms under various strain rates were analyzed. Based on the Dynamic Materials Model (DMM) theory, hot processing maps at true strains of 0.2, 0.4, 0.6, and 0.8 were constructed. And the applicability of the Prasad and Murty instability criteria to this new steel was compared. The results indicate that different recrystallization mechanisms operate at different strain rates. Compared to the commonly used Prasad criterion, the Murty instability criterion aligns better with the experimental observations for this steel. The optimal hot processing window for this new steel is identified as a temperature range of 1100–1150 °C with strain rates of 0.1–1 s^−1^ and 10 s^−1^, which is consistent with the microstructure of the deformed specimens. This work offers fundamental insights into the microstructural evolution, which is essential for optimizing the hot-working properties of next-generation high-strength stainless steels.

## 1. Introduction

Aircraft components face significant challenges regarding corrosion cracking during service, particularly in harsh marine environments over extended periods [1]. Due to their superior combination of mechanical properties and environmental corrosion resistance, high-strength stainless steels are preferred materials for key aerospace components [2,3,4,5].

To ensure superior comprehensive mechanical properties and corrosion resistance, elements such as Cr, Ni, Ti, Al, and Cu are typically added to high-strength stainless steels. The high alloy content leads to better high-temperature deformation resistance and a relatively narrow processing window for dynamic recrystallization (DRX) during hot deformation. Reported data indicate that in the temperature range of 900–1150 °C and strain rate range of 0.01–10 s^−1^, the flow stress of low-alloy high-strength steel 300 M is approximately 30–230 MPa, with DRX occurring between 1000 and 1150 °C [6]. For 30CrMnSiNi2A steel exhibits, the flow stresses is approximately 50–240 MPa in the range of 920–1120 °C and 0.01–10 s^−1^, with DRX occurring between 1020 and 1070 °C [7]. In comparison, Custom450 high-strength stainless steel shows flow stress of approximately 50–310 MPa under similar conditions (900–1150 °C, 0.01–10 s^−1^), with a DRX range of 1050–1150 °C [8]. It has been reported that a 1900 MPa-grade ultra-high strength stainless steel shows flow stress of approximately 75–350 MPa in the range of 950–1150 °C and 0.01–10 s^−1^, along with a DRX interval of 1050–1150 °C [9]. This comparison reveals that high-alloy high-strength stainless steels generally exhibit higher hot deformation resistance and narrower DRX temperature ranges compared to low-alloy high-strength steels.

Furthermore, plastic instability phenomena such as deformation twinning and localized shear bands can occur during hot deformation of high-strength stainless steels. The hot processing map theory, developed based on the Dynamic Materials Model (DMM), is an important tool for analyzing and predicting plastic instability during hot working, playing a significant role in microstructure and property control. It has recently become a research focus in advanced plastic processing and widely applied and validated in studies concerning hot forming mechanisms and microstructural evolution in various advanced materials, including steels [10,11], titanium alloys [12], superalloys [13], and metal matrix composites [14]. However, different instability criteria proposed within the DMM framework can yield significantly different or even contradictory results due to variations in their theoretical foundations, formulations, and physical interpretations [15,16]. Therefore, the suitability of different plastic instability criteria varies for different materials. For instance, Aneta et al. [15] studied the hot deformation behavior of 4340 steel via isothermal compression tests and optimized its hot working parameters using processing maps based on the Prasad criterion. Di et al. [16] established processing maps for Cr5 die steel based on the Prasad criterion within the parameter range of 1100–1300 °C and 0.01–10 s^−1^, investigating the effects of deformation temperature and strain rate on DRX. Xiong et al. [17] constructed processing maps for Ti-22Al-25Nb alloy using the Murty criterion, identifying two peak power dissipation efficiency (*η*) regions of 51% and 56% at 940 °C/0.001 s^−1^ and 1060 °C/0.001 s^−1^, respectively, associated with lamellar spheroidization and recrystallization phenomena. Zhan et al. [18] studied the hot deformation behavior of AA6016/AA7050/AA6016 laminated composites using both Prasad and Murty criteria, finding that the Prasad criterion more accurately identified the safe and unstable processing regions for the composite compared to the Murty criterion. Wang et al. [19] established hot processing maps for a 1900 MPa grade ultra-high strength stainless steel and comprehensively compared the suitability of the Prasad, Murty, Gegel, and Alexander-Malas (A-M) instability criteria based on metallographic observations of compressed specimens, concluding that the Murty criterion better matched the actual behavior of the high-strength stainless steel and discussed the mathematical relationships and physical meanings between the different criteria.

In industrial production, large-sized high-alloy steel ingots are highly susceptible to plastic instability during forging, leading to microstructural defects such as wedge cracks, adiabatic shear bands, deformation twins, and voids [20]. Unlike traditional high-strength steels, the novel steel investigated in this study features a Cobalt-free and ultra-low carbon design (0.01 wt.%) with a tensile strength reaching 1700 MPa. To improve cost-effectiveness and processing efficiency, this study investigates the hot deformation behavior of this new steel and explores the influence of strain rate on the dynamic recrystallization mechanism. Meanwhile, the applicability of the Prasad and Murty instability criteria is compared to determine the optimal hot working process window.

## 2. Materials and Methods

The steel sample was prepared using a double vacuum melting process consisting of vacuum induction melting (VIM) followed by vacuum arc remelting (VAR). The specific chemical composition is 0.01 wt.% C, 11.5 wt.% Cr, 10.5 wt.% Ni, 2.3 wt.% Mo, 0.3 wt.% Ti, and 1.8 wt.% Al. After homogenization treatment, the ingot was forged into a round bar with a diameter of Φ 150 mm, followed by a preparatory heat treatment. Specimens for hot compression testing were taken from the 1/2 radius position of the bar. Axial hot compression tests were conducted on a Gleeble-3800 thermomechanical simulator (Dynamic Systems Inc., Beijing, China). Cylindrical specimens measuring Φ 8 mm × 15 mm were heated to 1150 °C at a rate of 10 °C/s, held for 5 min, and then cooled to the target deformation temperature at 10 °C/s. Deformation temperatures (*T*) were set to 900, 950, 1000, 1050, 1100, and 1150 °C. Strain rates ($\dot{\varepsilon }$) were 0.01, 0.1, 1, and 10 s^−1^. True strain (*ε*) levels were set to 0.2, 0.4, 0.6, and 0.8. After deformation, specimens were immediately water-quenched to preserve the high-temperature deformed microstructure. The original microstructure and the hot compression process route are shown in Figure 1.

After sectioning, grinding, and polishing, the compressed specimens were etched using a 10% potassium permanganate sulfuric acid aqueous solution (composition: 10 mL H_2_SO_4_ + 90 mL H_2_O + 1 g KMnO_4_). Microstructure observation was performed using an OLYMPUS GX53 optical microscope (Olympus Corporation, Beijing, China) (OM), and analysis was conducted using OLYCIA m3 image analysis software (Olympus Corporation, Beijing, China, 25.3.7.1). Specimens for EBSD observation were also ground and mechanically polished, followed by electrolytic polishing. Observation was carried out using a JEOL JSM-7200F scanning electron microscope (JEOL Ltd., Beijing, China), with a step size of 0.65 μm and a scan area of 320 μm × 320 μm.

## 3. Results

### 3.1. Stress–Strain Curves

The true stress–strain curves of the steel under different deformation conditions are shown in Figure 2. It can be observed that at a constant strain rate, the flow stress decreases with increasing deformation temperature. This is because higher temperatures enhance dislocation mobility, allowing dislocations to overcome barriers more easily through climb or glide, thereby reducing the critical shear stress of the material [21]. At a constant deformation temperature, the flow stress increases with increasing strain rate. This is due to the shorter deformation time and accelerated dislocation multiplication rate at higher strain rates. Figure 2a,b and Figure 2c,d reveal two main types of stress–strain curves. At strain rates of 0.01 s^−1^ and 0.1 s^−1^, the curves exhibit distinct peak stresses (as shown in Figure 2a,b). In contrast, at strain rates of 1.0 s^−1^ and 10 s^−1^, the curves do not show obvious peak stresses (as shown in Figure 2c,d). The occurrence of peak stress is generally associated with dynamic recrystallization during hot deformation [22,23,24]. In the initial stage of hot deformation, work hardening dominates, causing a rapid increase in flow stress. With increasing strain, dynamic recovery or dynamic recrystallization occurs in the steel. When dynamic recrystallization occurs, the stress–strain curve typically shows a distinct peak stress. When only dynamic recovery occurs, the softening effect of recovery reaches a dynamic balance with work hardening, and no obvious peak stress appears on the curve.

### 3.2. Microstructure

During deformation, temperature, strain rate, and true strain are key factors affecting grain size and nucleation mechanisms. The microstructural changes in Figure 3 clearly show the influence of different deformation conditions on grain size. At a deformation temperature of 900 °C and true strains of 0.2–0.4, the original austenite grains are compressed (Figure 3a1,a2,b1,b2). When the true strain reaches 0.6–0.8 (Figure 3a3,a4,b3,b4), fine dynamically recrystallized grains appear at the original austenite grain boundaries. Due to the low deformation temperature, the DRX fraction is small, and dynamic recovery is the primary softening mechanism. At a deformation temperature of 1050 °C and a true strain of 0.2, the original austenite grains are compressed (Figure 3c1,d1). When the true strain reaches 0.4, fine DRX grains can be observed at the original austenite grain boundaries (Figure 3c2,d2). As the true strain increases, the DRX fraction continues to increase. When the true strain reaches 0.8, the DRX fraction exceeds 90% (Figure 3c3,c4,d3,d4). At a deformation temperature of 1100 °C and a true strain of 0.2, fine DRX grains are already observable at the original austenite grain boundaries (Figure 3e1,f1). With increasing true strain, the DRX fraction increases continuously. When the true strain reaches 0.8, complete dynamic recrystallization occurs (Figure 3e2–e4,f2–f4). Based on this analysis, it is evident that under constant strain rate and true strain conditions, both the DRX fraction and recrystallized grain size increase with increasing deformation temperature. Under constant temperature and true strain, an increase in strain rate leads to a higher DRX fraction but a smaller recrystallized grain size. This is because high strain rates promote rapid dislocation multiplication, significantly increasing dislocation density and stored energy, which provides more nucleation sites for DRX. Simultaneously, limited time for grain boundary migration results in finer recrystallized grains. Some studies suggest that higher strain rates can cause continuous dynamic recrystallization (CDRX) to replace discontinuous dynamic recrystallization (DDRX) as the dominant softening mechanism [25,26,27].

In the deformation temperature range of 900–950 °C, due to the low temperature, only partial DRX occurs regardless of the strain rate range (low: 0.01–0.1 s^−1^; high: 1–10 s^−1^), and dynamic recovery is the main softening mechanism. Consequently, the stress–strain curves do not exhibit distinct peaks. When the deformation temperature range is 1000–1150 °C, the DRX fraction increases, becoming the primary softening mechanism. Therefore, within the strain rate range of 0.01–0.1 s^−1^, the stress–strain curves show obvious peak stresses (Figure 2a,b). Comparing Figure 3c,e and Figure 3d,f, it can be revealed that under constant true strain, the DRX fraction increases with increasing strain rate. However, within the parameter range of 1000–1150 °C and 1–10 s^−1^, the stress–strain curves do not show distinct peaks (Figure 2c,d). To clarify this phenomenon, EBSD analysis was performed on the experimental steel.

To further analyze local orientation changes under different deformation conditions, Kernel Average Misorientation (KAM) maps with a maximum misorientation of 5° were generated based on EBSD data, as shown in Figure 4. Blue areas indicate grains with small local misorientations, corresponding to regions that have undergone DRX, where internal misorientation is lower than in deformed grains. These regions are mainly distributed near original grain boundaries. At deformation temperatures of 900 °C and 1050 °C, compared to a strain rate of 0.01 s^−1^, the steel exhibits a higher DRX fraction at 10 s^−1^ (more blue areas in Figure 4b,d. The variation in grain size can be found in Supplementary Table S1), while the accumulated dislocation density within the non-recrystallized original austenite grains is also higher (more yellow areas in Figure 4b,d). The average local misorientation is 2.00° and the high-angle grain boundary (HAGB) fraction is 34.1% for the condition T = 900 °C/*ε* = 0.8/$\dot{\varepsilon }\;$= 0.01s^−1^. For T = 1050 °C/*ε* = 0.8/$\dot{\varepsilon }$= 0.01 s^−1^, the values are 1.57° and 38.3%. For T = 900 °C/*ε* = 0.8/$\dot{\varepsilon }\;$= 10 s^−1^, they are 2.02° and 34.4%. For T = 1050 °C/*ε* = 0.8/$\dot{\varepsilon }\;$= 10 s^−1^, they are 1.64° and 54.7%. At 900 °C, only dynamic recovery occurred, and the microstructures at different strain rates are similar, but the high strain rate (10 s^−1^) produced slightly more recrystallized grains. At 1050 °C, DRX occurred. The HAGB fraction at the high strain rate is significantly greater than at the low strain rate (0.01 s^−1^), while the local misorientation is also higher. This indicates that the high strain rate promotes the occurrence of DRX while also facilitating rapid dislocation multiplication. Restricted grain boundary migration leads to increased dislocation density and stored energy, explaining the absence of a distinct peak stress in the stress–strain curve during DRX under high strain rates. The microstructural evolution follows a consistent trend across the full temperature range; additional data for the omitted conditions (e.g., T = 1150 °C) are provided in Supplementary Figure S1, confirming the complete DRX behavior at high temperatures.

To further analyze the orientation changes in recrystallized grains, Grain Orientation Spread (GOS) maps were plotted, as shown in Figure 5. Blue regions represent grains that have undergone DRX, while green regions represent the non-recrystallized parent phase. At low strain rates, recrystallized grains show larger orientation spreads and relatively random orientations, exhibiting typical discontinuous recrystallization characteristics (Figure 5a). At high strain rates, numerous low-angle grain boundaries (LAGBs) extend into the grains, and the orientations of recrystallized grains are similar to the parent phase, showing typical continuous recrystallization characteristics (Figure 5b).

### 3.3. Hot Processing Maps

In the Dynamic Materials Model (DMM), the metal hot working is regarded as an energy dissipation system [28]. Taking forging as an example, this system includes a power source, a power storage unit, and a power dissipation unit. Power generated by the source is transmitted to the storage unit, then transferred via their interface to the dissipation unit, where it is finally dissipated through plastic deformation. The workpiece is considered a non-linear power dissipater. The total power input (*P*) is dissipated through two main processes: (1) Energy consumed by plastic deformation, denoted as *G* (content of power dissipation). Most of this energy converts to heat, while a small portion is stored as crystal defect energy. (2) Energy consumed by microstructural evolution during deformation, denoted as *J* (co-content of power dissipation). It represents power dissipation related to microstructural changes such as dynamic recovery, dynamic recrystallization, dislocation activity, phase/particle growth under dynamic conditions, spheroidization of acicular structures, phase transformations, etc.

According to DMM, the total power *P* absorbed by the workpiece during plastic deformation is given by $\sigma \dot{\varepsilon }$, expressed as

(1) $$P=\sigma \dot{\varepsilon }=G+J=\int_{0}^{\dot{\varepsilon }}\sigma d\dot{\varepsilon }+\int_{0}^{\sigma }\dot{\varepsilon }d\;\sigma ,$$

The proportion of these two energies is determined by the strain rate sensitivity index (*m*) of the material at a given deformation temperature and strain:

(2) $$\frac{dJ}{dG}=\frac{\dot{\varepsilon }d\sigma }{\sigma d\dot{\varepsilon }}=\frac{d(ln\sigma )}{d(ln\dot{\varepsilon })}\approx \frac{∆log\sigma }{∆log\dot{\varepsilon }}=m$$

Under certain deformation temperature and strain conditions, the relationship between flow stress (σ) and strain rate ($\dot{\varepsilon }$) can be described dynamically as

(3) $$\sigma =K{\dot{\varepsilon }}^{m}$$

where K is the flow stress at a strain rate of 1 s^−1^, and m is the strain rate sensitivity index.

Then,

(4) $$\frac{dJ}{dG}=\frac{\dot{\varepsilon }d\sigma }{\sigma d\dot{\varepsilon }}=\frac{d(ln\sigma )}{d(ln\dot{\varepsilon })}\approx \frac{∆log\sigma }{∆log\dot{\varepsilon }}=m$$

where $K^{*}={\left(1/K\right)}^{1/m}$.

Integrating yields:

(5) $$J=\left[\frac{m}{m+1}\right]\sigma \cdot \varepsilon$$

When the material is in an ideal linear dissipation state (*m* = 1), *J* reaches its maximum value $J_{max}=\frac{\sigma \dot{\varepsilon }}{2}=\frac{P}{2}$.

The parameter reflecting the power dissipation characteristics is the efficiency of power dissipation (*η*), defined as the ratio of energy dissipated by microstructural evolution to the ideal linear dissipation energy:

(6) $$\eta =\frac{J}{J_{max}}$$

(7) $$\eta =\frac{J}{J_{max}}=\frac{J}{P/2}=\frac{2(P-G)}{P}=2\left(1-\frac{G}{P}\right)=2[1-\frac{1}{\sigma \dot{\varepsilon }}\int_{0}^{\dot{\varepsilon }}\sigma d\dot{\varepsilon }]$$

The physical meaning of *η* is the relative rate of internal entropy production during hot working, characterizing the dissipative microstructure under different processing temperatures and strain rates. It varies with deformation temperature and strain rate. A 3D plot or corresponding contour map of *η* is called the power dissipation map. The strain rate sensitivity *m* is derived from the relationship between flow stress and strain rate during deformation, representing the slope of the ln*σ* vs. ln$\dot{\varepsilon }$ curve. Under the investigated conditions, these curves rarely have a constant slope, and *m* often varies with strain rate. To derive the *m* value at any specific strain rate, a cubic spline function [29] was used to fit the curves for optimal fitting, with the results shown in Figure 6.

Common deformation mechanisms during hot deformation include dynamic recovery, dynamic recrystallization, superplasticity, wedge cracking, void formation, intergranular cracking, prior particle boundary cracking, and flow instability. Different deformation mechanisms correspond to different power dissipation efficiencies. Safe deformation mechanisms like dynamic recovery and dynamic recrystallization typically have high *η* values. However, a high *η* value does not always indicate good workability, as mechanisms like cracking and void formation can also exhibit high *η*. Therefore, instability criteria are needed to distinguish stable from unstable regions during hot deformation.

Based on Ziegler’s principle of maximum entropy production rate, Prasad et al. [30] assumed m to be constant regardless of strain rate and proposed a flow instability criterion as shown in Equation (8):

(8) $${\xi \left(\dot{\varepsilon }\right)}_{K-P}=\frac{\ln\left(\frac{m}{m+1}\right)}{\ln\dot{\varepsilon }}+m<0$$

Considering that m is not constant, Murty et al. [31] proposed a flow instability criterion applicable to any stress–strain curve, as shown in Equation (9):

(9) $${\xi \left(\dot{\varepsilon }\right)}_{M-R}=2m-\eta <0$$

Hot processing maps at a true strain of 0.8, constructed according to the different instability criteria, are shown in Figure 7. The unstable regions are mainly concentrated in the high strain rate range. The difference is that under the Prasad criterion, Region 1 is predicted as stable, while Regions 4 and 5 are predicted as unstable. Based on actual metallographic observations (Figure 8a–e corresponding to Regions 1–5), plastic instability occurred in Region 1, whereas no plastic instability was observed in Regions 4 and 5 (corresponding to 1100–1150 °C/1 s^−1^). This indicates that for the experimental steel, the Murty criterion is more consistent with reality. Therefore, the Murty criterion was used for constructing the hot processing maps in this study.

As shown in Figure 7b, the power dissipation efficiency *η* increases with increasing deformation temperature and decreasing strain rate, i.e., *η* increases from the top-left to the bottom-right of the contour map. It is generally considered that DRX occurs when *η* > 0.3. The region of high *η* values is located at 1070–1150 °C/0.06–1.82 s^−1^. However, a high *η* value does not strictly equate to the occurrence of DRX. In Region 7 (1050–1150 °C/10 s^−1^, high temperature, high strain rate), the *η* value is 0.14–0.17, theoretically suggesting no DRX. However, combining this with Figure 8h–i reveals that complete DRX occurred in this region, indicating that DRX can also occur in low *η* regions. The instability regions are primarily concentrated in the high strain rate area, as shown in Figure 7b. Region 6 represents the hot processing window at 0.8 true strain, and its metallographic results in Figure 8f–i all show complete DRX. Overall, the Murty criterion demonstrates better adaptability, and the hot processing maps effectively reflect the actual microstructural evolution. The processing window at 0.8 true strain is 1100–1150 °C/0.1–1 s^−1^ and 1100–1150 °C/10 s^−1^.

Based on extensive examination of the quenched microstructures after deformation, a microstructure map evolving with deformation conditions was established, as shown in Figure 7c. DRV indicates dynamic recovery is the dominant softening mechanism; C-DRX indicates continuous dynamic recrystallization is dominant; D-DRX indicates discontinuous dynamic recrystallization is dominant; ‘Complete DRX’ indicates full dynamic recrystallization occurred in the marked region; ‘Grain Growth’ indicates that not only does DRX occur, but significant grain growth also happens at high temperatures and low strain rates.

## 4. Conclusions

Ours findings provide fundamental insights into the microstructural evolution during hot deformation, which not only elucidates the strain-rate-dependent DRX mechanisms but also establishes reliable processing maps. These findings are expected to accelerate the development and industrial application of next-generation high-strength stainless steels.

(1) Under the conditions of deformation temperatures of 900–1150 °C and strain rates of 0.01–10 s^−1^ (Figure S2), the flow stress curves of the new ultra-high strength stainless steel exhibit two forms. At low strain rates, the curves show a peak stress. With increasing strain rate, work hardening intensifies, the decreasing trend of flow stress slows, and the ratio of peak stress to steady-state stress gradually decreases to 1, where no peak stress is observed. However, dynamic recrystallization can occur under both curve types.
(2) At low strain rates, the dynamic recrystallization mechanism in the new ultra-high strength stainless steel is primarily discontinuous dynamic recrystallization (DDRX), involving nucleation and growth. At high strain rates, the mechanism shifts to continuous dynamic recrystallization (CDRX), where lattice rotation caused by shear stress leads to the transformation of low-angle grain boundaries into high-angle grain boundaries, directly forming recrystallized grains.
(3) Based on the hot processing maps, the instability regions for the new ultra-high strength stainless steel are concentrated at low deformation temperatures and high strain rates. With increasing true strain, the η value increases, and the degree of dynamic recrystallization becomes more complete. The optimal hot processing window for this new ultra-high strength stainless steel is 1100–1150 °C with strain rates of 0.1–1 s^−1^ and 1100–1150 °C with a strain rate of 10 s^−1^.

## Figures and Tables

**Figure 1:**
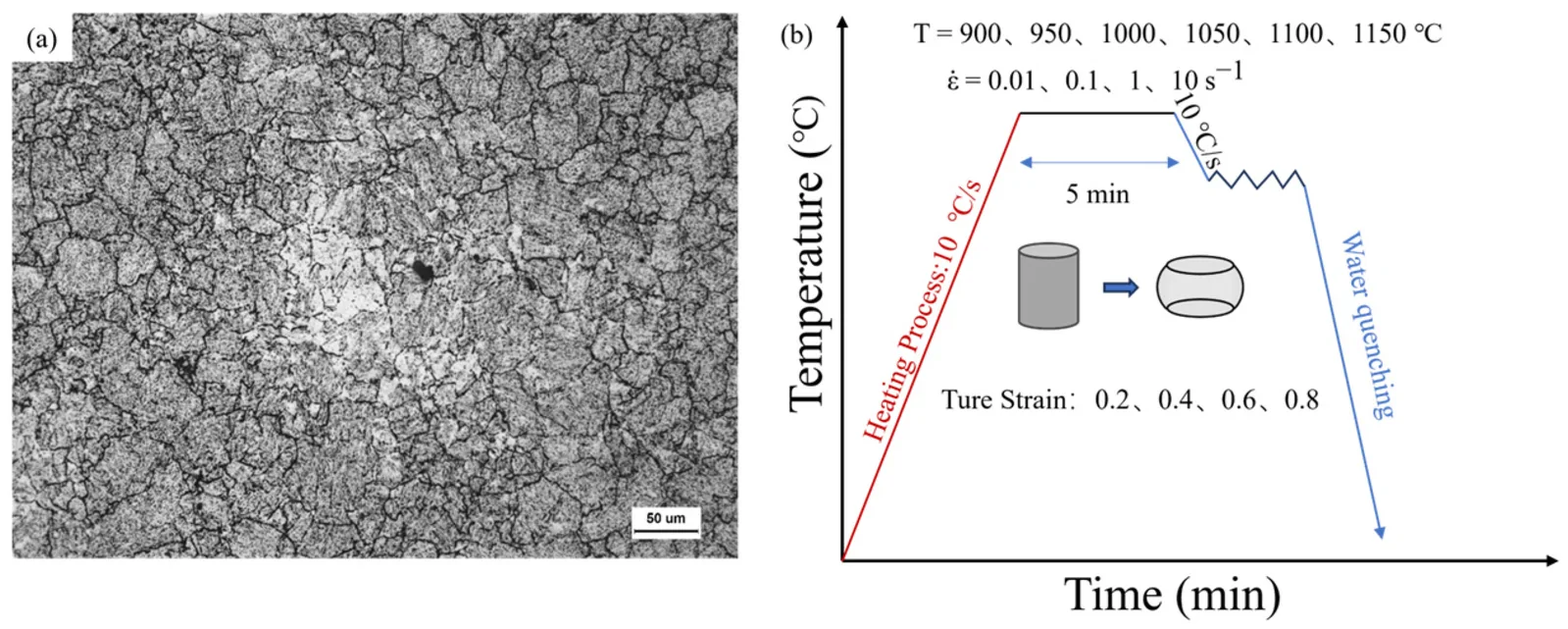
(**a**) Initial Microstructure, (**b**) Schematic diagram of the isothermal hot compression test process.

**Figure 2:**
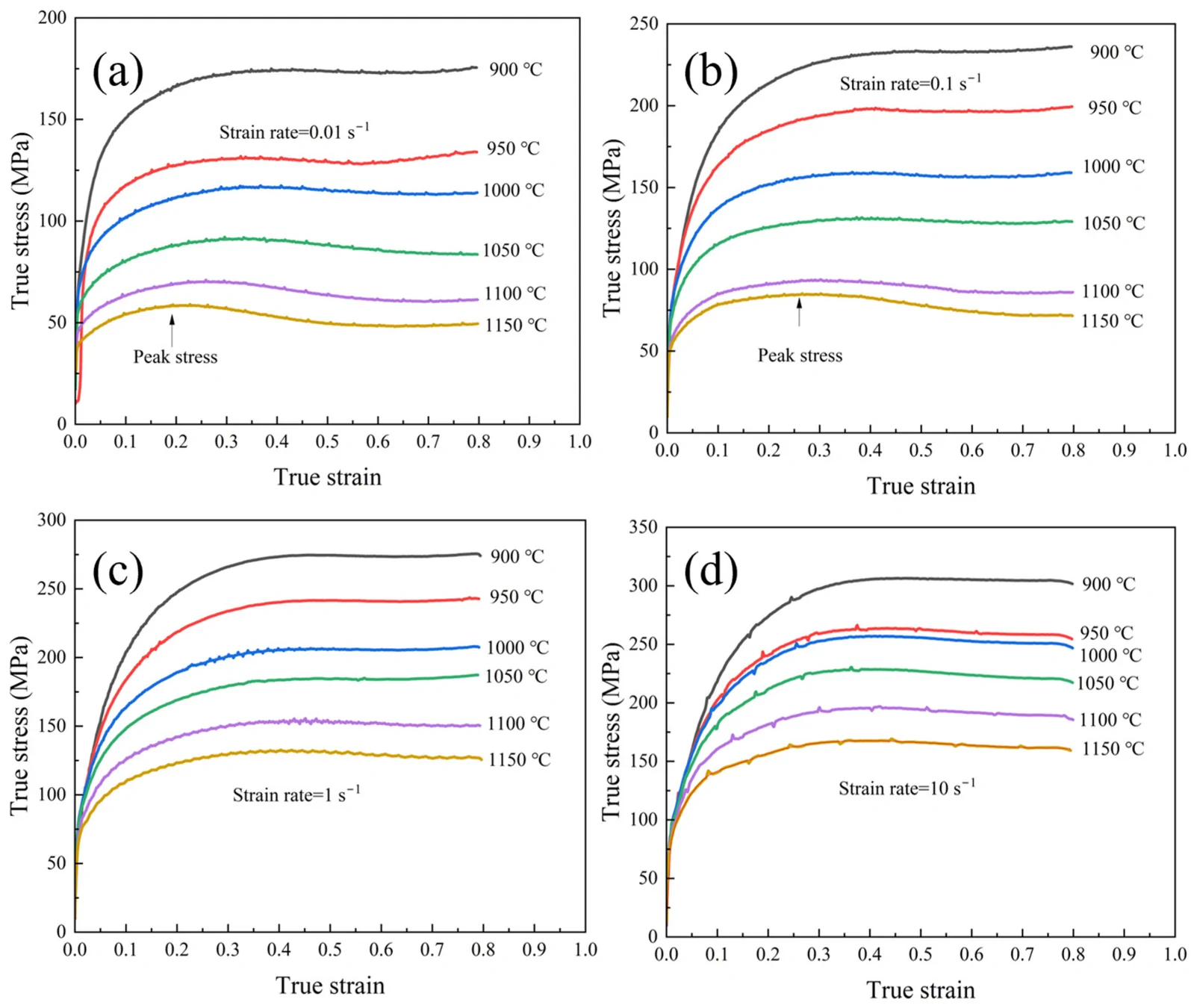
True stress–strain curves under different strain rates ($\dot{\varepsilon }$): (**a**) $\overset{\cdot }{\varepsilon }$ = 0.01 s^−1^, (**b**) $\overset{\cdot }{\varepsilon }$ = 0.1 s^−1^, (**c**) $\overset{\cdot }{\varepsilon }$ = 1 s^−1^, (**d**) $\overset{\cdot }{\varepsilon }$ = 10 s^−1^.

**Figure 3:**
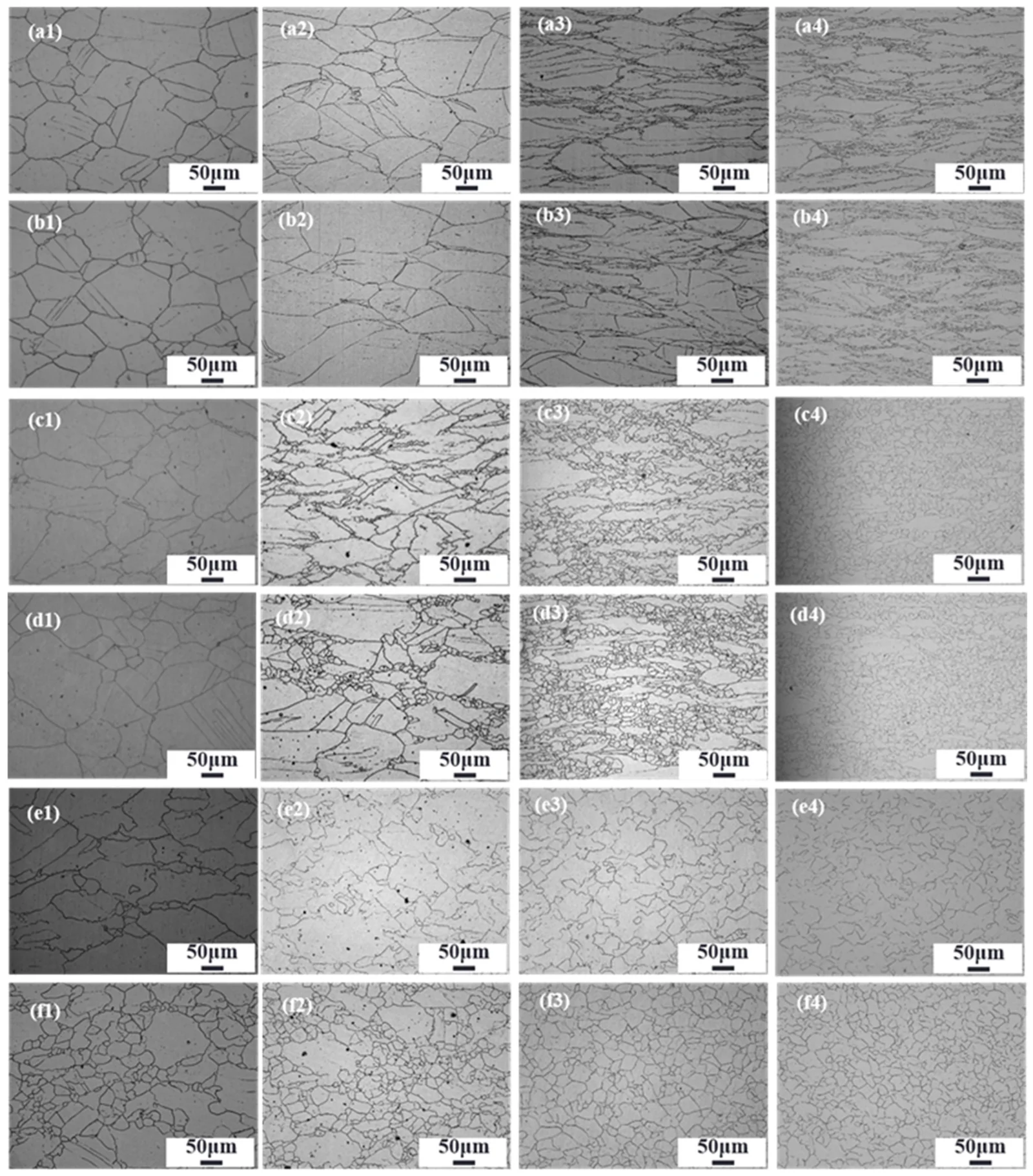
Microstructures of the original austenite grain under different hot deformation processes: (**a1**) *T* = 900 °C/*ε* = 0.2/$\dot{\varepsilon }$ = 0.01 s^−1^, (**a2**) *T* = 900 °C/*ε* = 0.4/$\dot{\varepsilon }\;$= 0.01 s^−1^, (**a3**) *T* = 900 °C/*ε* = 0.6/$\dot{\varepsilon }$ = 0.01 s^−1^, (**a4**) *T* = 900 °C/*ε* = 0.8/$\dot{\varepsilon }$ = 0.01 s^−1^, (**b1**) *T* = 900 °C/*ε* = 0.2/$\dot{\varepsilon }$ = 10 s^−1^, (**b2**) *T* = 900 °C/*ε* = 0.4/$\dot{\varepsilon }\;$= 10 s^−1^, (**b3**) *T* = 900 °C/*ε* = 0.6/$\dot{\varepsilon }\;$= 10 s^−1^, (**b4**) *T* = 900 °C/*ε* = 0.8/$\dot{\varepsilon }\;$= 10 s^−1^, (**c1**) *T* = 1000 °C/*ε* = 0.2/$\dot{\varepsilon }\;$= 0.01 s^−1^, (**c2**) *T* = 1000 °C/*ε* = 0.4/$\dot{\varepsilon }\;$= 0.01 s^−1^, (**c3**) *T* = 1000 °C/*ε* = 0.6/$\dot{\varepsilon }\;$= 0.01 s^−1^, (**c4**) *T* = 1000 °C/*ε* = 0.8/$\dot{\varepsilon }\;$= 0.01 s^−1^, (**d1**) *T* = 1000 °C/*ε* = 0.2/$\dot{\varepsilon }\;$= 10 s^−1^, (**d2**) *T* = 1000 °C/*ε* = 0.4/$\dot{\varepsilon }\;$= 10 s^−1^, (**d3**) *T* = 1000 °C/*ε*= 0.6/$\dot{\varepsilon }\;$= 10 s^−1^, (**d4**) *T* = 1000 °C/*ε* = 0.8/$\dot{\varepsilon }\;$= 10 s^−1^, (**e1**) *T* = 1100 °C/*ε* = 0.2/$\dot{\varepsilon }$ = 0.01 s^−1^, (**e2**) *T* = 1100 °C/*ε* = 0.4/$\dot{\varepsilon }\;$= 0.01 s^−1^, (**e3**) *T* = 1100 °C/*ε* = 0.6/$\dot{\varepsilon }\;$= 0.01 s^−1^, (**e4**) *T* = 1100 °C/*ε* = 0.8/$\dot{\varepsilon }\;$= 0.01 s^−1^, (**f1**) *T* = 1100 °C/*ε* = 0.2/$\dot{\varepsilon }\;$= 10 s^−1^, (**f2**) *T* = 1100 °C/*ε* = 0.4/$\dot{\varepsilon }\;$= 10 s^−1^, (**f3**) *T* = 1100 °C/*ε* = 0.6/$\dot{\varepsilon }\;$= 10 s^−1^, (**f4**) *T* = 1100 °C/*ε* = 0.8/$\dot{\varepsilon }\;$= 10 s^−1^.

**Figure 4:**
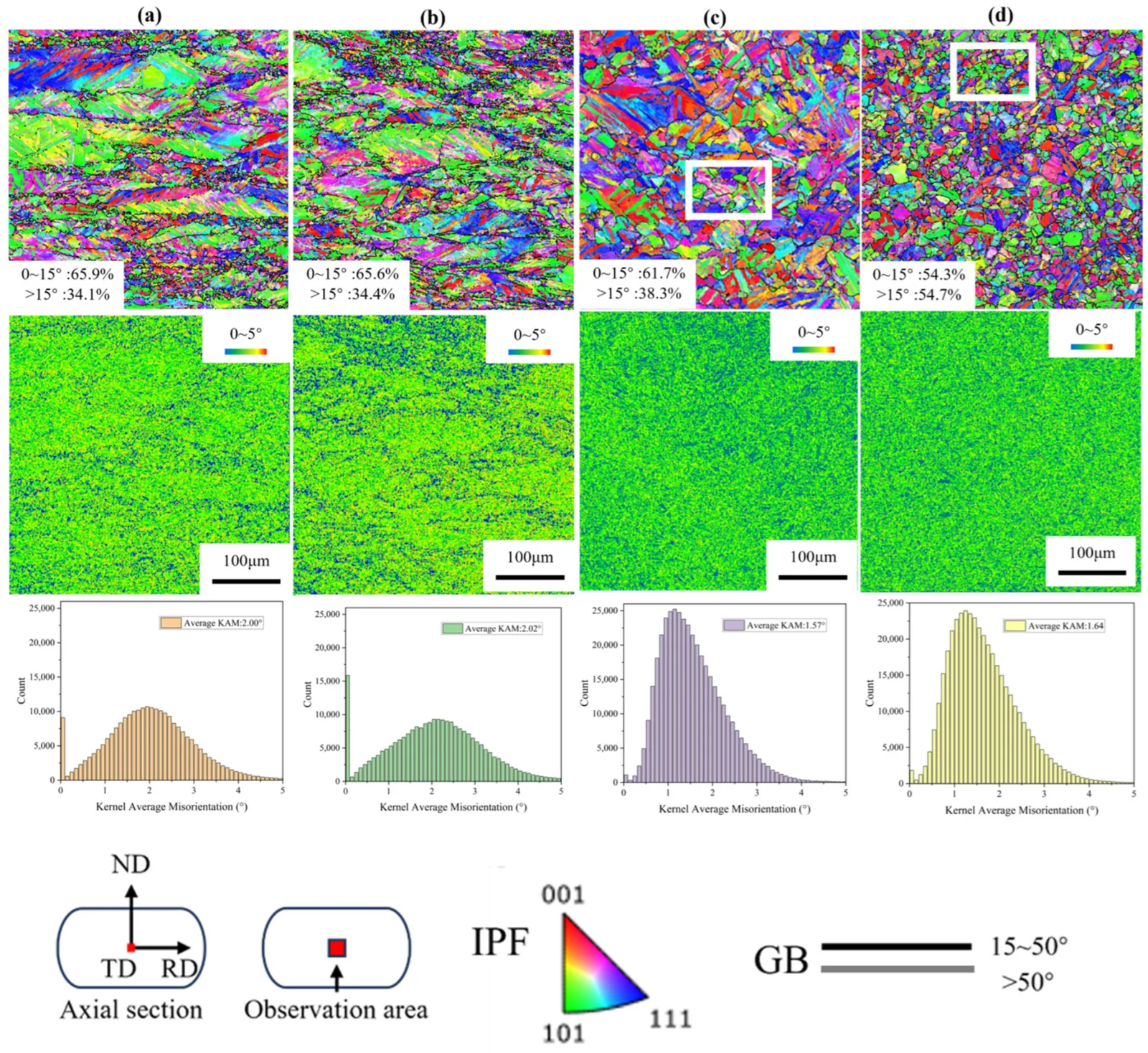
IPF maps, GB maps, and KAM analysis of microstructure under different deformation conditions via EBSD: (**a**) *T* = 900 °C/*ε* = 0.8/$\dot{\varepsilon }$ = 0.01 s^−1^, (**b**) *T* = 900 °C/*ε* = 0.8/$\dot{\varepsilon }$= 10 s^−1^, (**c**) *T* = 1050 °C/*ε* = 0.8/$\dot{\varepsilon }\;$= 0.01 s^−1^, (**d**) *T* = 1050 °C/*ε* = 0.8/$\dot{\varepsilon }\;$= 10 s^−1^.

**Figure 5:**
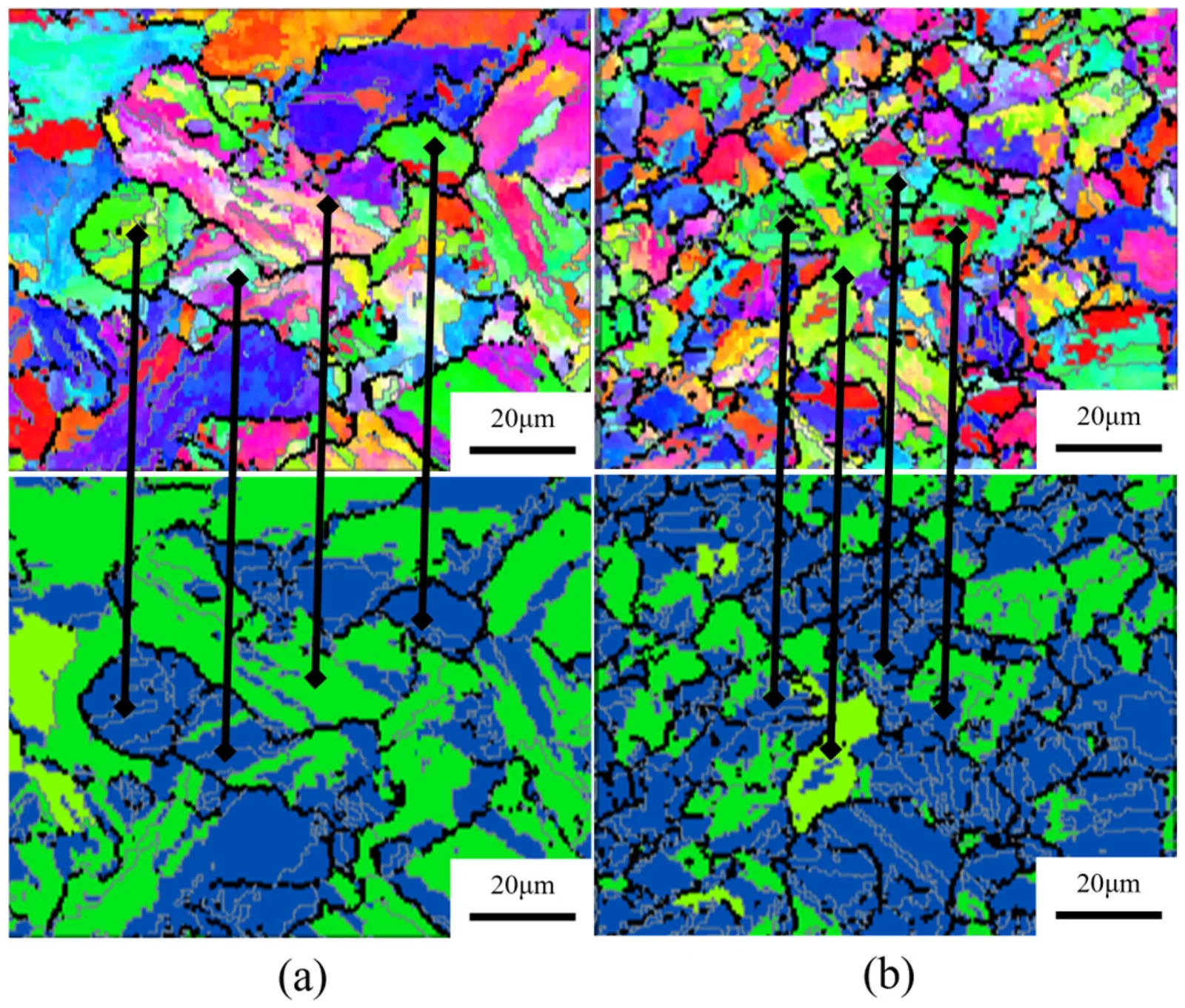
Magnified views and recrystallization map analysis of the white rectangular areas in Figure 4: (**a**) *T* = 1050 °C/*ε* = 0.8/$\dot{\varepsilon }$ = 0.01 s^−1^, (**b**) *T* = 1050 °C/*ε* = 0.8/$\dot{\varepsilon }\;$= 10 s^−1^.

**Figure 6:**
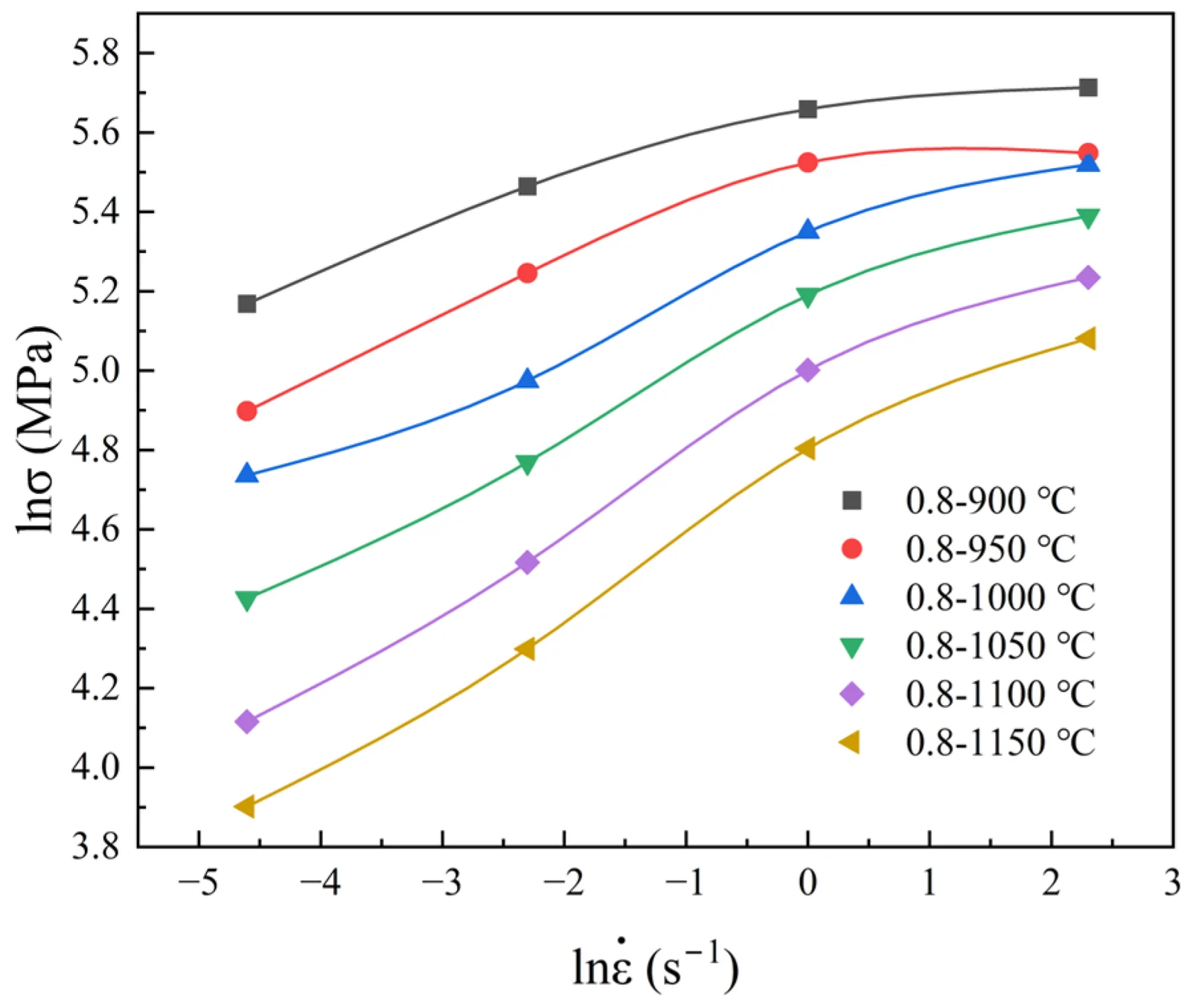
Relationship between ln*σ* and ln$\dot{\varepsilon }$.

**Figure 7:**
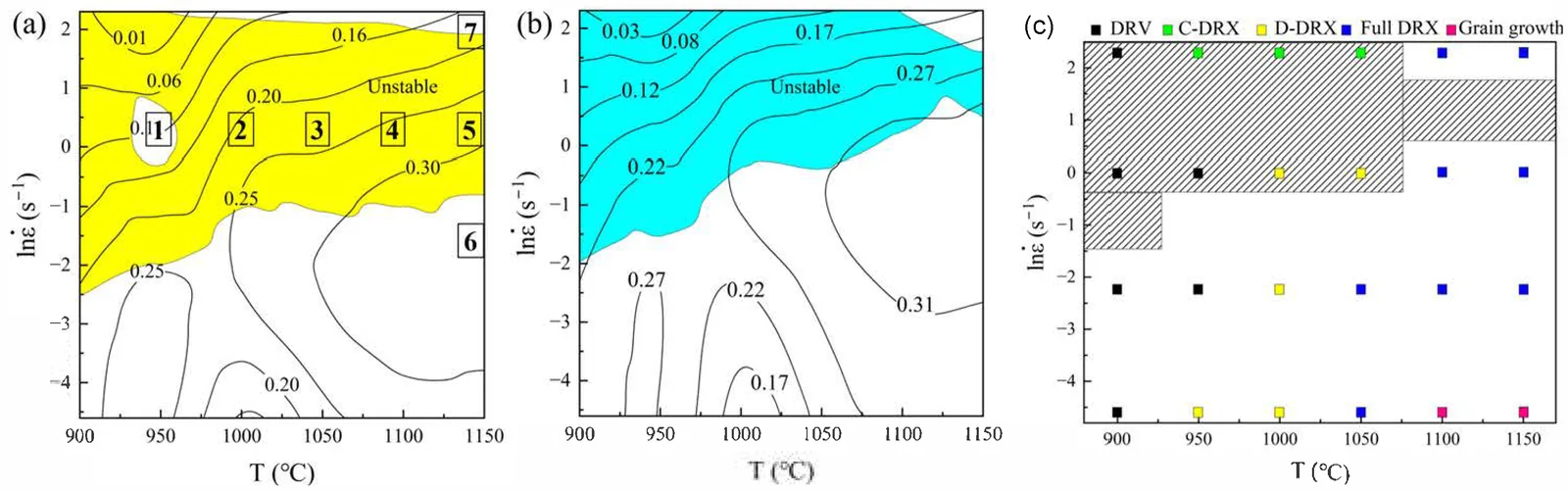
Hot processing maps at a true strain of 0.8: (**a**) Prasad criterion, (**b**) Murty criterion, (**c**) Microstructure map under different deformation conditions.

**Figure 8:**
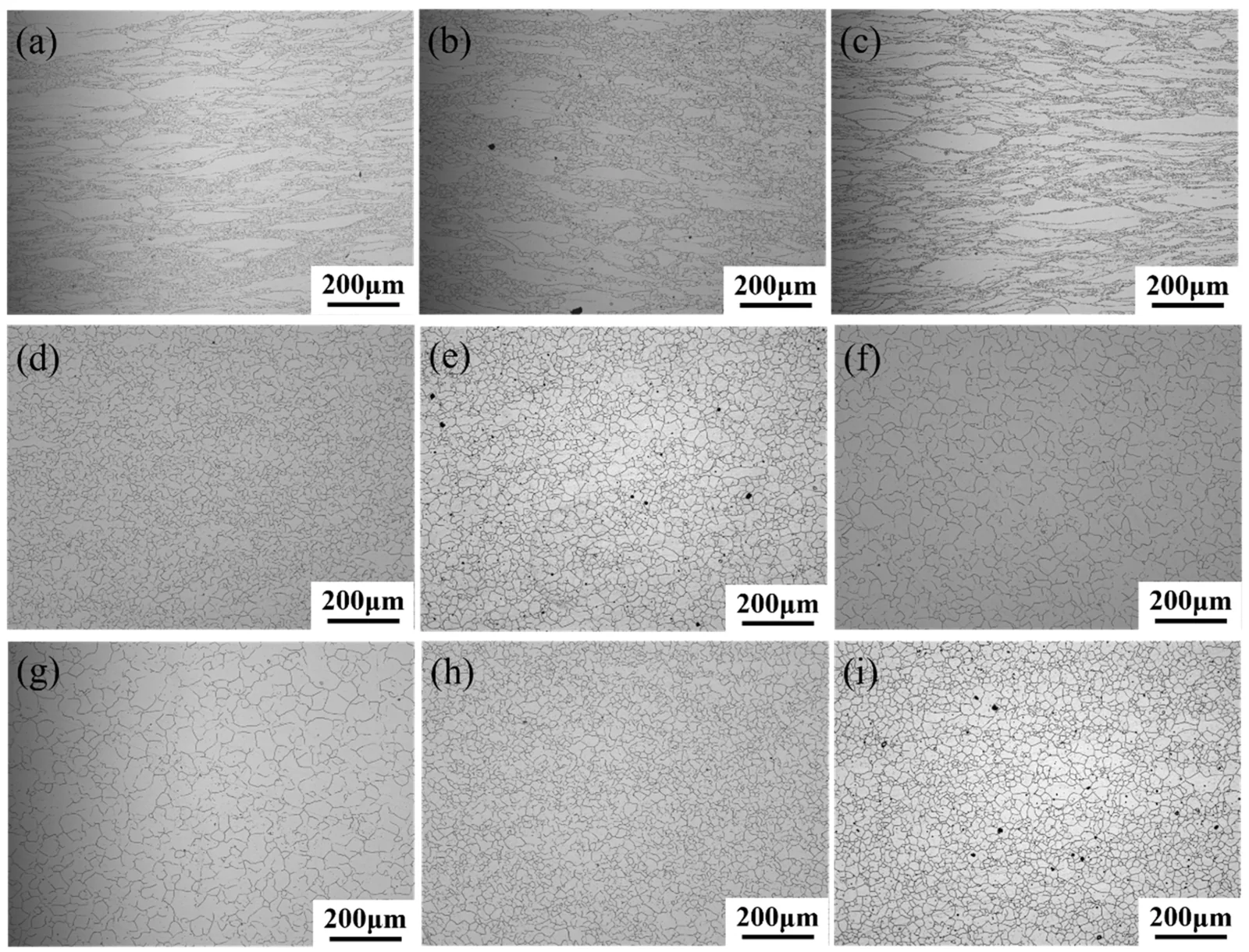
The grain morphology of original austenite under different hot deformation conditions: (**a**) T = 950 °C/$\dot{\varepsilon }$ = 1 s^−1^, (**b**) T = 1000 °C/$\dot{\varepsilon }\;$= 1 s^−1^, (**c**) T = 1050 °C/$\dot{\varepsilon }$ = 1 s^−1^, (**d**) T = 1100 °C/$\dot{\varepsilon }\;$= 1 s^−1^, (**e**) T = 1150 °C/$\dot{\varepsilon }\;$= 1 s^−1^, (**f**) T = 1100 °C/$\dot{\varepsilon }\;$= 0.1 s^−1^, (**g**) T = 1150 °C/$\dot{\varepsilon }\;$= 0.1 s^−1^, (**h**) T = 1100 °C/$\dot{\varepsilon }\;$= 10 s^−1^, (**i**) T = 1150 °C/$\dot{\varepsilon \;}$= 10 s^−1^.

## Data Availability

The original contributions presented in this study are included in the article/Supplementary Materials. Further inquiries can be directed to the corresponding authors.

## Supplementary Materials

The following supporting information can be downloaded at: https://www.mdpi.com/article/10.3390/ma19194088/s1, Figure S1: IPF maps, GB maps, and KAM analysis of microstructure under different deformation conditions via EBSD: (a) T = 1150 °C/ε = 0.8/$\overset{\cdot }{\varepsilon }$ = 0.01 s^−1^, (b) T = 1150 °C/ε = 0.8/$\overset{\cdot }{\varepsilon }$ = 10 s^−1^; Figure S2: Microstructures of the original austenite grain under different hot deformation processes: (a1) T = 900 °C/ε = 0.8/$\overset{\cdot }{\varepsilon }$ = 0.01 s^−1^, (a2) T = 900 °C/ε = 0.8/$\overset{\cdot }{\varepsilon }$ = 0.1 s^−1^, (a3) T = 900 °C/ε = 0.8/$\overset{\cdot }{\varepsilon }$ = 1 s^−1^, (a4) T = 900 °C/ε = 0.8/$\overset{\cdot }{\varepsilon }$ = 10 s^−1^, (b1) T = 950 °C/ε = 0.8/$\overset{\cdot }{\varepsilon }$ = 0.01 s^−1^, (b2) T = 950 °C/ε = 0.4/$\overset{\cdot }{\varepsilon }$ = 0.1 s^−1^, (b3) T = 950 °C/ε = 0.8/$\overset{\cdot }{\varepsilon }$ = 1 s^−1^, (b4) T = 950 °C/ε = 0.8/$\overset{\cdot }{\varepsilon }$ = 10 s^−1^, (c1) T = 1000 °C/ε = 0.8/$\overset{\cdot }{\varepsilon }$ = 0.01 s^−1^, (c2) T = 1000 °C/ε = 0.8/$\overset{\cdot }{\varepsilon }$ = 0.1 s^−1^, (c3) T = 1000 °C/ε = 0.8/$\overset{\cdot }{\varepsilon }$ = 1 s^−1^, (c4) T = 1000 °C/ε = 0.8/$\overset{\cdot }{\varepsilon }$ = 10 s^−1^, (d1) T = 1050 °C/ε = 0.8/$\overset{\cdot }{\varepsilon }$ = 0.01 s^−1^, (d2) T = 1050 °C/ε = 0.8/$\overset{\cdot }{\varepsilon }$ = 0.1 s^−1^, (d3) T = 1050 °C/ε = 0.8/$\overset{\cdot }{\varepsilon }$ = 1 s^−1^, (d4) T = 1050 °C/ε = 0.8/$\overset{\cdot }{\varepsilon }$ = 10 s^−1^, (e1) T = 1100 °C/ε = 0.8/$\overset{\cdot }{\varepsilon }$ = 0.01 s^−1^, (e2) T = 1100 °C/ε = 0.8/$\overset{\cdot }{\varepsilon }$ = 0.1 s^−1^, (e3) T = 1100 °C/ε = 0.8/$\overset{\cdot }{\varepsilon }$ = 1 s^−1^, (e4) T = 1100 °C/ε = 0.8/$\overset{\cdot }{\varepsilon }$ = 10 s^−1^, (f1) T = 1150 °C/ε = 0.8/$\overset{\cdot }{\varepsilon }$ = 0.01 s^−1^, (f2) T = 1150 °C/ε = 0.8/$\overset{\cdot }{\varepsilon }$ = 0.1 s^−1^, (f3) T = 1150 °C/ε = 0.8/$\overset{\cdot }{\varepsilon }$ = 1 s^−1^, (f4) T = 1150 °C/ε = 0.8/$\overset{\cdot }{\varepsilon }$ = 10 s^−1^; Table S1: Avg. Grain Size (μm).
