# Supplementary material for: Study on Rheological Behavior and Hot Processing Maps of a Novel Ultra-Low Carbon High-Strength Stainless Steel
Source: Materials (Basel). 2026 Sep 24;19(19):4088. doi: 10.3390/ma19194088 (PMC13665888; doi:10.3390/ma19194088)
Supplement: Supplementary file 1 [file materials-19-04088-s001.zip › materials-4067336-supplementary.pdf]

Table S1. Avg. Grain Size (μm)

T=1000 °C/ε= 0.2/$\dot{\varepsilon}$=10 s^{-1}	T=1000 °C/ε= 0.4/$\dot{\varepsilon}$=10 s^{-1}	T= 1000 °C/ε= 0.6/$\dot{\varepsilon}$=0.01 s^{-1}	T= 1000 °C/ε= 0.8/$\dot{\varepsilon}$=10 s^{-1}
64.32	44.76	25.47	14.54

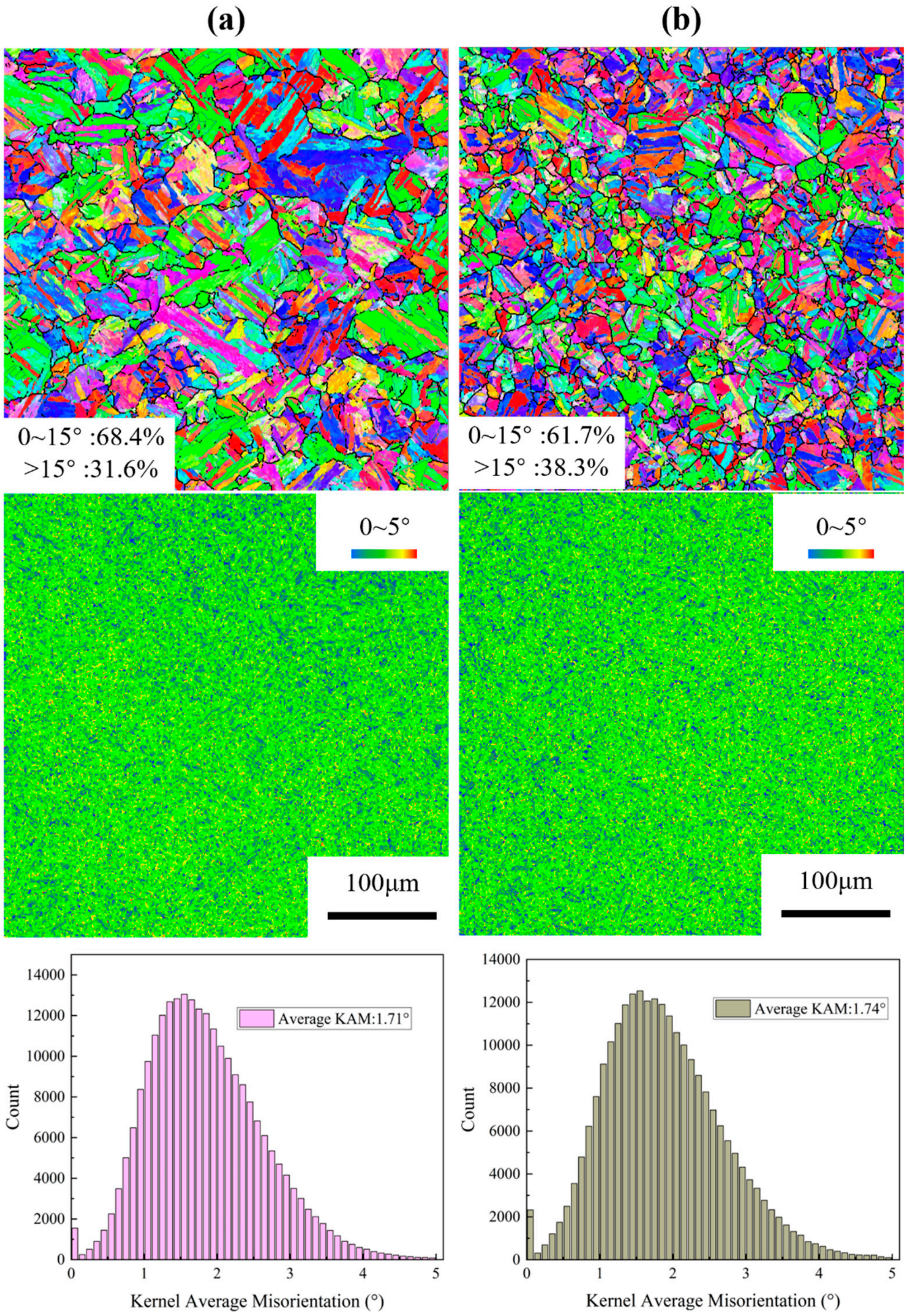

Figure S1. IPF maps, GB maps, and KAM analysis of microstructure under different deformation conditions via EBSD: (a) T=1150 °C/ε= 0.8/$\dot{\varepsilon}$=0.01 s^{-1}, (b)

T=1150 °C/ε= 0.8/$\dot{\varepsilon}$=10 s^{-1}

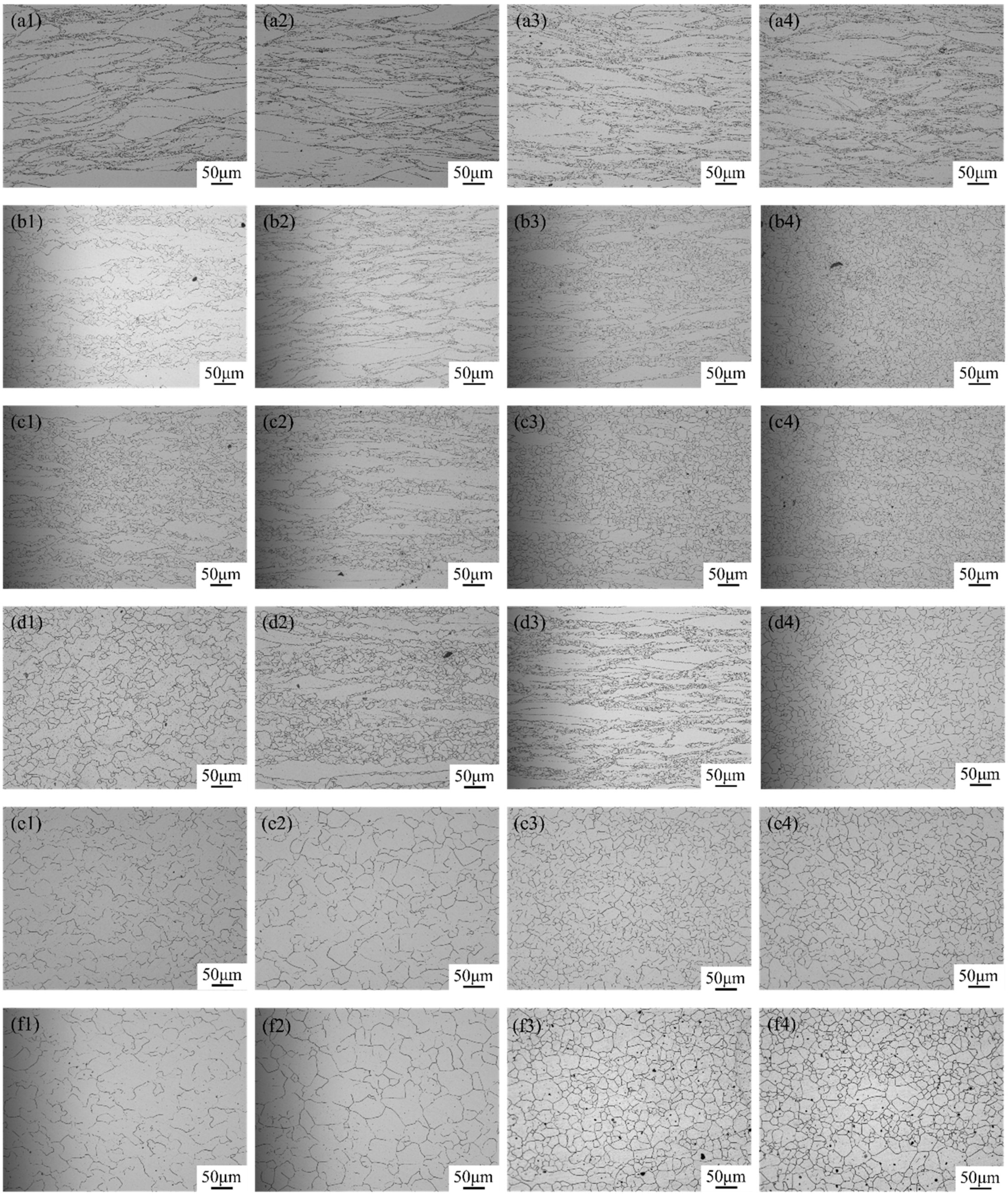

Figure S2. Microstructures of the original austenite grain under different hot deformation processes: (a1) T=900 °C/ε= 0.8/$\dot{\varepsilon}$=0.01 s^{-1}, (a2) T=900 °C/ε= 0.8/$\dot{\varepsilon}$=0. 1 s^{-1}, (a3) T=900 °C/ε= 0.8/$\dot{\varepsilon}$=1 s^{-1}, (a4) T=900 °C/ε= 0.8/$\dot{\varepsilon}$=10 s^{-1}, (b1) T=950 °C/ε= 0.8/$\dot{\varepsilon}$=0.01 s^{-1}, (b2) T=950 °C/ε= 0.4/$\dot{\varepsilon}$=0.1 s^{-1}, (b3) T=950 °C/ε= 0.8/$\dot{\varepsilon}$=1 s^{-1}, (b4) T=950 °C/ε= 0.8/$\dot{\varepsilon}$=10 s^{-1}, (c1) T=1000 °C/ε= 0.8/$\dot{\varepsilon}$=0.01 s^{-1}, (c2) T=1000 °C/ε= 0.8/$\dot{\varepsilon}$=0.1 s^{-1}, (c3) T=1000 °C/ε= 0.8/$\dot{\varepsilon}$=1 s^{-1}, (c4) T=1000 °C/ε= 0.8/$\dot{\varepsilon}$=10 s^{-1}, (d1) T=1050 °C/ε= 0.8/$\dot{\varepsilon}$=0.01 s^{-1}, (d2) T=1050 °C/ε= 0.8/$\dot{\varepsilon}$=0.1 s^{-1}, (d3) T=1050 °C/ε= 0.8/$\dot{\varepsilon}$=1 s^{-1}, (d4) T=1050 °C/ε= 0.8/$\dot{\varepsilon}$=10 s^{-1}, (e1) T=1100 °C/ε= 0.8/$\dot{\varepsilon}$=0.01 s^{-1}, (e2) T=1100 °C/ε= 0.8/$\dot{\varepsilon}$=0.1 s^{-1}, (e3) T=1100 °C/ε= 0.8/$\dot{\varepsilon}$=1 s^{-1}, (e4) T=1100 °C/ε= 0.8/$\dot{\varepsilon}$=10 s^{-1}, (f1) T=1150 °C/ε= 0.8/$\dot{\varepsilon}$=0.01 s^{-1}, (f2) T=1150 °C/ε= 0.8/$\dot{\varepsilon}$=0.1 s^{-1}, (f3)

T=1150 °C/ε= 0.8/$\dot{\varepsilon}$=1 s^{-1}，(f4) T=1150 °C/ε= 0.8/$\dot{\varepsilon}$=10 s^{-1}.
